# The Janus kinase 1/2 inhibitor ruxolitinib in COVID-19 with severe systemic hyperinflammation

**DOI:** 10.1038/s41375-020-0891-0

**Published:** 2020-06-09

**Authors:** F. La Rosée, H. C. Bremer, I. Gehrke, A. Kehr, A. Hochhaus, S. Birndt, M. Fellhauer, M. Henkes, B. Kumle, S. G. Russo, P. La Rosée

**Affiliations:** 1 Medizinische Fakultät Carl Gustav Carus, Technische Universität Dresden, Dresden, Germany; 2 Lungenzentrum Donaueschingen, Schwarzwald-Baar-Klinikum, Villingen-Schwenningen, Germany; 3 Klinik für Innere Medizin IV, Schwarzwald-Baar-Klinikum, Villingen-Schwenningen, Germany; 4 Klinik für Innere Medizin II, Universitätsklinikum Jena, Jena, Germany; 5 Apotheke/Institut für Klinische Pharmazie, Schwarzwald-Baar-Klinikum, Villingen-Schwenningen, Germany; 6 Klinik für Innere Medizin II, Hämatologie, Onkologie, Immunologie, Infektiologie und Palliativmedizin, Schwarzwald-Baar-Klinikum, Villingen-Schwenningen, Germany; 7 Klinik für Akut- und Notfallmedizin, Schwarzwald-Baar-Klinikum, Villingen-Schwenningen, Germany; 8 Klinik für Anästhesiologie, Intensiv-, Notfall- und Schmerzmedizin, Villingen-Schwenningen, Germany; 9 Medizinische Fakultät, Universität Göttingen, Göttingen, and Fakultät für Gesundheit, Universität Witten/Herdecke, Witten, Germany; 10 Medizinische Fakultät der Friedrich-Schiller-Universität Jena, Universitätsklinikum Jena, Jena, Germany

**Keywords:** Inflammatory diseases, Cytokines, Translational research

## Abstract

A subgroup of patients with severe COVID-19 suffers from progression to acute respiratory distress syndrome and multiorgan failure. These patients present with progressive hyperinflammation governed by proinflammatory cytokines. An interdisciplinary COVID-19 work flow was established to detect patients with imminent or full blown hyperinflammation. Using a newly developed COVID-19 Inflammation Score (CIS), patients were prospectively stratified for targeted inhibition of cytokine signalling by the Janus Kinase 1/2 inhibitor ruxolitinib (Rux). Patients were treated with efficacy/toxicity guided step up dosing up to 14 days. Retrospective analysis of CIS reduction and clinical outcome was performed. Out of 105 patients treated between March 30th and April 15th_,_ 2020, 14 patients with a CIS ≥ 10 out of 16 points received Rux over a median of 9 days with a median cumulative dose of 135 mg. A total of 12/14 patients achieved significant reduction of CIS by ≥25% on day 7 with sustained clinical improvement in 11/14 patients without short term red flag warnings of Rux-induced toxicity. Rux treatment for COVID-19 in patients with hyperinflammation is shown to be safe with signals of efficacy in this pilot case series for CRS-intervention to prevent or overcome multiorgan failure. A multicenter phase-II clinical trial has been initiated (NCT04338958).

## Introduction

The novel coronavirus (SARS-CoV-2) pandemic is a global health crisis. A 1–5% mortality rate affecting particularly comorbid patients is currently observed [1]. The disease has been designated COVID-19, an acronym for “coronavirus disease 2019”. To date, efficacy of antiviral drugs explored in COVID-19 awaits confirmation [2, 3].

While most people with COVID-19 develop mild or uncomplicated illness, ~14% develop severe disease requiring hospitalization and oxygen support and 5% require admission to an intensive care unit. In severe cases, COVID-19 can be complicated by acute respiratory distress syndrome (ARDS), sepsis, and/or multiorgan failure (MOF) [4]. Recent multivariable analysis confirmed older age, higher Sequential Organ Failure Assessment (SOFA) score and D-Dimer >1 µg/L on admission associated with higher mortality [5].

Severe and critically affected patients develop bilateral viral pneumonia, which is categorized as hypersensitivity pneumonitis [6]. Autopsy lung tissue shows diffuse infiltration of hyperactivated T-cells, as does T-cell typing in the peripheral blood [7]. Chinese COVID-19 series report hyperinflammation governed by pro-inflammatory cytokines in particular in patients with dismal outcome indicating a significant role of cytokine release for tissue damage and multiorgan failure [4, 8, 9]. Management of COVID-19 patients requires hospitals to setup COVID-19 specific infrastructure to prevent uncontrolled transmissions, establish high-end multidisciplinary knowledge teams to provide a continuum of care from emergency room to intensive care unit (ICU) and into weaning and rehabilitation facilities.

Ruxolitinib (Rux; Jakavi®) is a potent and selective inhibitor of Janus kinases (JAK) 1 and 2, with modest to marked selectivity against tyrosine kinase (TYK)2 and JAK3, respectively. Rux is currently approved in the European Union (EU) for the treatment of primary myelofibrosis (PMF), post-polycythemia vera (PV) or post-essential thrombocythemia myelofibrosis and for the treatment of adult patients with PV [10]. Key to Rux efficacy is its broad anti-inflammatory activity against the myeloproliferative neoplasm (MPN) inherent cytokine storm with pro-inflammatory IL-1, IL-6, IL-8, IL-12, TNF-α, IFNγ, VEGF, TGFβ, FGF, PDGF, GM-CSF, and G-CSF cytokines/growth factors [11]. Rux is highly effective in *off-label* indications, where cytokine release plays a central role for pathogenesis: Graft versus host disease (GvHD) and hemophagocytic lymphohistiocytosis (HLH) [12–15]. Rux doses ranging from 5 mg bid (GvHD) up to 25 mg bid (HLH) were successfully used without signs of overt toxicity. As both conditions go along with a significant risk of viral or bacterial reactivation, safe immunomodulation without safety signals is of particular interest in light of unknown mechanisms of SARS-CoV-2 viral clearance [16–18].

Many patients with severe respiratory disease due to COVID-19 have features consistent with cytokine release syndrome (CRS) [19, 20]. Due to increased activation of the JAK/STAT pathway, it is postulated that JAK-inhibitors might have a useful role in treating these patients [21, 22].

## Methods

### Study design

This is a monocentric retrospective chart analysis on consecutive patients admitted to the Schwarzwald–Baar–Klinikum Villingen-Schwenningen, Germany, with severe COVID-19 and a multidisciplinary board decision on specific medical treatment. Assessment of systemic inflammation was done using a trial specific newly developed clinical inflammation score, named COVID Inflammation Score (CIS) (Table 1). The score was developed through integration of published patient characteristics from the Chinese case series [5, 23, 24]. Patients achieving the threshold score value of ≥10 (out of max. 16 score points) without clinical signs of sepsis (procalcitonin (PCT) negative, no uncontrolled active infection) were deemed at high risk for systemic inflammation based on cytokine release and evaluable for Rux treatment. First patient treated was March 30th, 2020. Date of last treatment initiation was April 15th, 2020 with cut-off for follow-up on April 21st, 2020 (patient #14, day 7). Severity was defined if any of the following conditions was met: (1) respiratory rate ≥ 30 breaths/min; (2) SpO_2_ ≤ 93% while breathing ambient air; (3) PaO_2_/FiO_2_ ≤ 300 mmHg. Critical COVID-19 was diagnosed if any of (1) respiratory failure requiring mechanical ventilation, (2) shock, (3) combined with other organ failure requiring admission to ICU occurred.

**Table 1:** COVID hyperinflammation score (≥10 of 16 threshold for inclusion).

Points	
Chest-X-ray/Chest-CT consistent w/hypersensitivity pneumonitis	**3**
CRP > 20 × ULN	**2**
Ferritin > 2 × ULN	**2**
Triglycerides > 1.5 × ULN	**1**
IL-6 > 3 × ULN	**1**
Fibrinogen > ULN	**1**
Leukocytes > ULN	**1**
Lymphopenia < 1.1/nL	**2**
Fever > 38.5 °C	**2**
Coagulation disorder	**1**
- DIC (D-Dimer > ULN)	
- PTT > ULN	

*ULN* Upper limit of normal, *DIC* Disseminated Intravascular Coagulation, *PTT* Partial thromboplastin time.

### Ruxolitinib treatment

Rux was provided by the hospital pharmacy as 15 mg tablets. Based on available prescription data on Rux and devoid of publicly available data on Rux in COVID-19, we decided on an intermediate dose between published trial results in GvHD (5 mg bid) and hemophagocytic lymphohistiocytosis (15 mg bid) and started treatment with 7.5 mg bid [12, 25]. Daily follow-up of efficacy and toxicity guided dosage with stepwise dose increase (15mg-0-7.5 mg; 15mg-0-15mg) at days 3, 5, or 7 by COVID-board decision was in place. Extended treatment duration in patients with clinical benefit and careful benefit-risk assessment was decided individually. Patients with active infections, severe hepatic impairment prior to systemic inflammation and underlying comorbidity with inherent survival probability <6 months were excluded. Recommendations for supportive and antiviral treatment were taken from the national COVID-19 guidelines [26].

### Efficacy and toxicity assessment

Efficacy was defined as achievement of 25% reduction in the CIS on day 7 compared to baseline. Radiologic response was taken from the X-ray/CT reports: “Deteriorated” compared with baseline was scored “3”, unchanged “2”, improved “1”, “resolved” was scored “0”. Ferritin response received gradual scoring for response assessment according to percent change of serum concentration compared to baseline: > 20% increase scored “2” (progression), +/− 20% scored “1” (unchanged), and > 20% decrease compared to baseline was scored 0 (response). Response for the reminder parameters was defined binary, i.e. reduction below the defined inflammation level was scored “0”. The clinical course of patients was assessed by the 7 point ordinal WHO scale at baseline, day 7 and day 15 as proposed by the WHO R&D blueprint (https://www.who.int/blueprint/priority-diseases/key-action/novel-coronavirus/en/). In adddition, the NEWS2-score to assess ICU-parameters for respiratory state and vital signs was assessed along days 0, 5, 7, and 15 (https://www.rcplondon.ac.uk/news/news2-and-deterioration-COVID-19). Toxicity was assessed looking at adverse events of special interest as provided in the Jakavi® prescription information (hematologic toxicity, liver toxicity) according to common toxicity criteria of adverse events version 5.0 (CTCAE 5.0).

#### Structured COVID-19 care algorithm (COVID-19-SOP)

The medical team caring for COVID-19 was structured into the respiratory/IMC/ICU core team (pneumologists/ICU care specialists), the regular care team for primary workup of patients with respiratory symptoms and fever and the immunomodulation team from Hematology/Immunology. The Schwarzwald–Baar–Klinikum (SBK) is a two campus General Academic Teaching Hospital of the University of Freiburg, allowing separation of COVID-19 patients in the Lung Center Campus Donaueschingen from the main campus (Villingen-Schwenningen). Special consideration was given COVID-19 ventilation management focusing on optimized non-invasive ventilation. This hospital wide respiratory management was developed to prevent patients from early intubation as the COVID-19-specific lung damage causes high mortality rates on invasive ventilation [27]. Patients received acetylsalicylic acid 500 mg, ascorbic acid 1000 mg, hydroxychloroquine 600 mg bid day 1, and 200 mg bid day 2–5, low molecular weight heparin providing therapeutic anticoagulation guided by D-Dimer levels and empiric antibiotic treatment. A defined COVID-lab sample was taken including extended coagulation parameters (PTT, D-Dimer, Fibrinogen), inflammation markers (C-reactive protein, ferritin, IL-6, sIL2-R, triglycerides) and routine emergency room lab tests including haematology. Daily COVID-19 board meetings were held with structured board reports. The CIS was calculated to aid treatment decisions regarding specific inflammation directed treatment with Rux. Short term corticosteroids at 2 mg/kg day 1–3 prednisolone was allowed based on individual comorbidity and disease severity aspects. Patients with excessive serum IL-6 levels >200 pg/ml and critical conditions were candidates for anti-IL-6 blockade by tocilizumab 400 mg single dose (patients #2, #13) according to recently discussed data on the WHO COVID-19 global literature database (https://search.bvsalud.org/global-literature-on-novel-coronavirus-2019-ncov/; accessed April 24th, 2020).

### Data analysis

Data were retrieved from patient charts and the electronic hospital information System Orbis (AGFA Health Care GmbH, Bonn, Germany). Systematic data acquisition for descriptive analysis was done using MS Excel (Microsoft, Redmond, WA, USA). Significance testing for CIS reduction as surrogate endpoint was done using Wilcoxon test for related samples using calculator on https://www.socscistatistics.com (two-sided, significance level 0.05).

## Results

### Patient characteristics

**Fig. 1:**
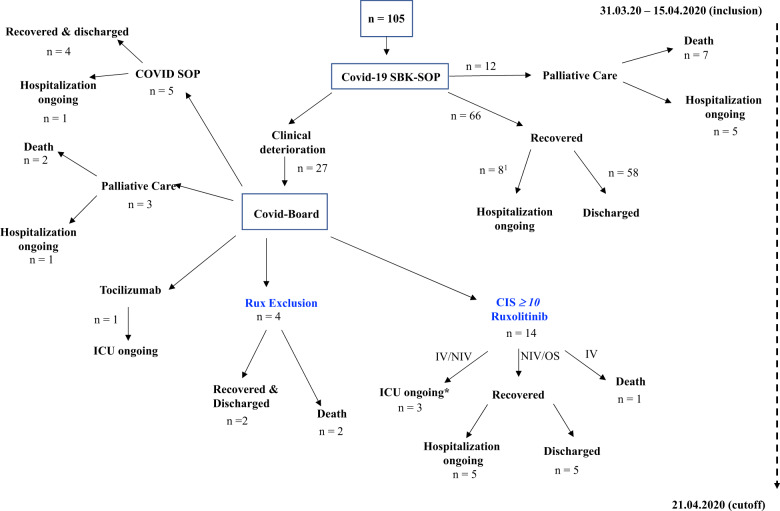
COVID-19 patient flow at Schwarzwald–Baar–Klinikum during March 31th and April 21st 2020. IV invasive ventilation, NIV non-invasive ventilation, OS oxygen support, *n = 1 Rux after Tocilizumab. ^1^Two patients started Ruxolitinib after data cutoff and recovered.

From March 30th to April 15th, 2020, *n* = 105 patients were hospitalized for COVID-19 treatment (Fig. 1). Patient characteristics are shown in Table 2 with a male predominance in the CIS ≥ 10 hyperinflammatory patient cohort. COVID-19 was confirmed via SARS-CoV-2-PCR positivity. A total of 66/105 (63%) patients stratified to standard of care (SOC) treatment recovered without further intervention. A total of 12/105 (11%) patients with primary stratification to palliative care died (*n* = 7) or were still hospitalized at data cut-off (*n* = 5). A total of 27/105 patients deteriorated on SOC and entered COVID-19 interdisciplinary board assessment after determination of the CIS (Table 1). Patients at high risk for hyperinflammation according to CIS and stratified to Rux treatment were 66 (55-81) years old with a male predominance (11/14) (Table 2). Ten out of 14 patients presented with fever >38.5 °C, the majority was on non-invasive ventilation (10/14), two were on oxygen support (3–4 l/min), one on high-flow oxygen (20 l/min) and one patient was admitted via inter-hospital transfer on invasive ventilation (FiO_2_ 90%). All patients presented with radiologic signs consistent with bilateral COVID-19 pneumonitis or COVID-19 associated ARDS. Arterial hypertension (11/14), smoking (9/14), hyperlipidemia (7/14), diabetes mellitus (5/14), and pre-existing lung disease (5/14) were the leading comorbidities. One patient was on postsurgical care after curative treatment for lung cancer with intrahospital CoV-2-infection and another patient on immunosuppression for vasculitis. A total of 5/14 patients presented with a respiratory rate > 25/min. The median NEWS2-score at baseline was 8.5 (4–16) indicating severe or critical COVID-19 in all patients stratified to Rux (Table 2).

**Table 2:** Baseline demographics and clinical characteristics.

Characteristics (total cohort)	Total (*N* = 105)			
Age, median (IQR)—years	65 (32-95)			
Male sex—no. (%)	58 (55)			
Coexisting conditions—no. (%)				
Diabetes mellitus	28 (27)			
Arterial hypertension	67 (64)			
Hyperlipidemia	21 (20)			
Cerebrovascular disease	5 (5)			
Cardiovascular disease	36 (34)			
Lung disease	34 (32)			
Malignant disease	16 (15)			
Immunosuppression	1 (1)			
Smoker/Ex-smoker	28 (27)			
**Characteristics (Rux-cohort)**	Total (*N* = 14)	Invasive ventilation (*N* = 1)	Non-invasive ventilation (*N* = 10)	Oxygen support (*N* = 3)
Age, median (IQR)—years	66 (55-81)	65	65 (55-81)	68 (64-70)
Male sex—no. (%)	11 (79)	1	7 (70)	3 (100)
Coexisting conditions—no. (%)				
Diabetes mellitus	5 (36)	1	4 (40)	0 (0)
Arterial hypertension	11 (79)	1	7 (70)	3 (100)
Hyperlipidaemia	7 (50)	1	3 (30)	3 (100)
Cerebrovascular disease	2 (14)	0	0 (0)	2 (67)
Cardiovascular disease	6 (43)	1	3 (30)	2 (67)
Lung disease	5 (36)	0	4 (50)	1 (33)
Malignant disease	1 (7)	0	0 (0)	1 (33)
Immunosuppression	1 (7)	0	1 (10)	0 (0)
Smoker/Ex-smoker	9 (64)	1	6 (60)	2 (67)
Fever ≥38.5 °C—no. (%)	10 (71)	0	8 (80)	2 (67)
Respiratory rate ≥25/min—no. (%)	5 (36)	1	2 (20)	2 (67)
Systolic blood pressure <90 mmHg - no. (%)	0 (0)	0	0 (0)	0 (0)
Oxygen-support category - no. (%)				
Invasive ventilation	1 (7)	1	–	–
Non-invasive ventilation	10 (71)	–	10 (100)^a^	–
High-flow oxygen support	1 (7)	–	–	1 (33)
Low-flow oxygen support	2 (14)	–	–	2 (67)
**NEWS2 score at baseline, median (IQR)**			8.5 (4-16)	
**Seven—point ordinal scale at baseline**				
4: Hospitalization, requiring supplemental oxygen—no. (%)			2 (14)	
5: Hospitalization, requiring HFNC or non-invasive mechanical ventilation—no. (%)			11 (79)	
6: Hospitalization, requiring ECMO, invasive mechanical ventilation, or both—no. (%)			1 (7)	

*HFNC* high-flow nasal cannula, *ECMO* extracorporeal membrane oxygenation.

#### COVID inflammation score (CIS) stratified medical treatment

Median days from onset of first symptoms to hospitalization and to Rux treatment were 9 (4–19) and 15.5 (5–24), respectively (Table 3). C-reactive protein (CRP) was highly elevated in the majority with 22.3 (1.6–67) fold upper limit of normal (ULN) (Table 4). Interleukin-6 (IL6) baseline values showed high variability with a median of 19 (3–282) fold ULN. Ferritin confirmed high inflammatory activity with 1585 ng/ml median elevation in conjunction with soluble interleukin-2 receptor (sIL2-R) positivity (1673 U/ml (994–4917)). In contrast, procalcitonin was normal in all but one patient. This single patient was deemed “sepsis uncertain” due to vasculitis-dependent PCT activation as consulted by the rheumatologist. Patients were not cytopenic except for anemia in individual patients (median Hb 12.9 g/dl (8.6–15.9)). Liver function was nearly normal at baseline with median alanine aminotransferase (ALT) and aspartate aminotransferase (AST) values sharply above ULN (Table 4).

**Table 3:** Patient clinical assessment and treatments received.

	Total (*N* = 14)
Treatment since hospitalization, no. (%)	
Invasive ventilation	3 (21)
Non-invasive ventilation	13 (93)
Renal-replacement therapy	1 (7)
Antibiotic agent	12 (86)
Hydroxychloroquine	13 (93)
Vasopressors	4 (29)
Tocilizumab	2 (14)
Glucocorticoid therapy	11 (79)
Days of glucocorticoid therapy, median (IQR)	3 (3–15)
Ruxolitinib dosage and therapy length	
Cumulative dosage, median (IQR)—mg	135 (52.5–285)
Length of treatment, median (IQR)—days	9 (5–17)
Days from illness onset to Ruxolitinib treatment, median (IQR)	15.5 (5–24)
Days from illness onset to hospitalization, median (IQR)	9 (4–19)^a^

^a^One patient suffered from in hospital SARS-CoV-2 transmission.

**Table 4:** Laboratory assessment.

Toxicity and Laboratory values—median (IQR)	Normal range	Baseline	Day 5	Day 7	Day 15
Serum Creatinine, mg/dl	0.5–1.1	1.01 (0.74–6.57)	0.89 (0.64–4.3)	0.82 (0.51–6.43)	0.92 (0.55–1.13)
total Bilirubin, mg/dl	0.2–1.1	0.5 (0.1–0.9)	0.5 (0.1–2.2)	0.45 (0.1–1.6)	0.65 (0.5–0.8)
AST, U/l	<50	54 (22–150)	66.5 (26–196)	56.5 (27–176)	37 (23–116)
ALT, U/l	<50	49.5 (21–157)	109 (27–299)	100 (34–415)	83 (36–221)
Triglycerides, mg/dl	<150	179 (116–512)	258·5 (114–432)	252 (171–311)	158.5 (95–263)
Triglycerides, x ULN		1.2 (0.8–3.4)	1.75 (0.8–2.9)	1.7 (1.1–2.1)	1 (0.6–1.8)
LDH, U/l	<250	456.5 (283–990)	355.5 (207–790)	333 (199–737)	444 (338–777)
Ferritin, ng/ml	15–400	1585 (498–6931)	1678.5 (306–3891)	1532 (274–3483)	1488 (1006–2958)
Ferritin, x ULN		4 (1.3–43.3)	4.2 (1.8–24.3)	3.8 (1.3–21.7)	3.7 (2.5–18.5)
PTT > ULN		0.9 (0.7–1.5)	0.9 (0.7–1.8)	0.9 (0.7–1.1)	0.8 (0.7–1.1)
Fibrinogen, x ULN		1.3 (0.7–2.5)	1.5 (0.7–2)	1.6 (0.9–2)	1.3 (0.8–1.6)
D-Dimer, x ULN		3.5 (1.1–21.8)	2.3 (1.1–22.9)	2.5 (0.8–19.5)	5.5 (0.5–8.6)
C reactive protein, x ULN		22.3 (1.6–67)	10.3 (1–56.7)	5.4 (0.7–51.9)	3.1 (0.3–48.7)
Procalcitonin ng/ml	<0.5	0.17 (0.02–3.79)	0.07 (0.02–1.21)	0.05 (0.02–1.16)	0.04 (0.02–0.43)
IL-6, x ULN		19 (3.1–282.1)	3.3 (0.4–179.4)	1.25 (0.2–119.4)	5.9 (2.1–11.2)
sIL2-R, U/ml	158–623	1673 (994–4917)	1340.5 (718–3568)	1052 (733–1877)	778 (687–1255)
Hematological laboratory					
White blood-cell count, /nl	4–10	8.3 (5–17)	10 (4.4–15.7)	8.4 (3.3–17.2)	8.2 (3.9–20.2)
White blood-cell count, x ULN		0.8 (0.5–1.7)	1 (0.4–1.6)	0.8 (0.3–1.9)	0.8 (0.4–2)
Platelet count, /nl	150–450	189.5 (105–397)	324.5 (124–850)	379.5 (109–937)	301.5 (223–540)
Neutophil count, /nl	1.4–6.5	6.14 (3.59–13.76)	6.96 (2.6–12.84)	5.94 (1.84–15.48)	3.94 (2.6–16.22)
Lymphocyte count, /nl	1.2–3.4	0.97 (0.52–2.15)	1.23 (0·71–3.7)	1.27 (0.51–3.54)	1.68 (0.82–3.09)
Hemoglobin, g/dl	12–18	12.9 (8.6–15.9)	12.3 (7.7–14.5)	12.2 (7.4–15.5)	10.2 (7.9–13.6)

**Fig. 2:**
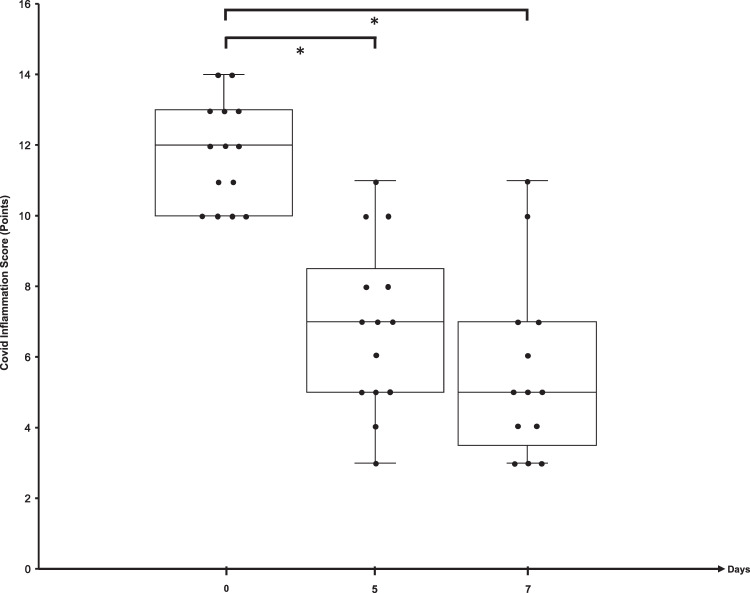
COVID inflammation score at baseline, day 5 and day 7 after Rux treatment initiation. Dots represent individual patient results. Median and IQR are provided by box plots. *p < 0.01.

The median CIS of patients stratified to CRS-targeted treatment was 12 (10–14) at baseline (Fig. 1). Treatment with starting dose of 7.5 mg Rux bid led to marked clinical control within days (patients #1, 3, 4, 6, 8, 9, 10, 11, 14) (Fig. 2). CIS reduction by 25% was achieved on day 5 and day 7 with a decline by 42% and 58%, respectively (Fig. 1, Table 5).

**Table 5:** Primary and secondary outcome results.

	Total (*N* = 14)			
CIS improvement Day 7 > 25%, no. (%)^a^	12 (86)			
Days of hospitalization, median (IQR)	18 (9–36)			
Intermediate care, no. (%)	6 (43)			
Days of Intermediate care, median (IQR)	5 (2–12)			
Intensive care, no. (%)	6 (43)			
Days of Intensive care, median (IQR)	18.5 (1–26)			
	Baseline (*N* = 14)	Day 5 (*N* = 14)	Day 7 (*N* = 14)	Day 15 (*N* = 11)
CIS improvement %, median (IQR)	–	42 (15–70)	58 (15–77)^b^	50 (15–54)^c^
NEWS2 scale, median (IQR)	8.5 (4–16)	4.5 (14–2)	4 (2–13)^b^	7 (3–13)^d^
7—point ordinal scale, no. (%)				
2—Not hospitalized, limitations on activities	0 (0)	1 (7)	1 (7)	5 (46)
4—Hospitalized, requiring supplemental oxygen	2 (14)	0 (0)	3 (21)	1 (9)
5—Hospitalized, on non-invasive ventilation or HFNC^e^	11 (79)	10 (71)	7 (50)	4 (36)
6—Hospitalization, requiring ECMO^f^, invasive mechanical ventilation, or both	1 (7)	3 (21)	3 (21)	1 (9)

^a^13 pts follow-up.

^b^13 pts follow-up.

^c^3 pts follow-up.

^d^4 pts follow-up.

^e^HFNC: High-flow nasal cannula.

^f^ECMO: Extracorporeal membrane oxygenation.

Three patients with insufficient response were dose escalated to 15 mg bid (day 16, patient #7), and 15mg-0-7.5 mg (patients #2 and #5). Patients #7 and #5 finally were consented to treatment limitation due to progressing multiorgan failure. Patient #7 (65 y/o) entered specific Rux treatment after emergency interhospital transfer at high level invasive ventilation (FiO_2_ 0.9) and was significantly comorbid (diabetes mellitus, arterial hypertension, coronary heart disease, smoking, hyperlipidemia). Patient #5 (70 y/o) suffered from significant pre-existing comorbidities (chronic obstructive lung disease, smoking, obesity, arterial hypertension). Patient #12 with elevated PCT at first COVID-board presentation (ongoing treatment of polyangiitis with pulmonary and renal affection; on azathioprine and chronic dialysis) was observed and treated according to SOC for sepsis. After rheumatology consult, the diagnosis sepsis without proven bloodstream infection was weighed against vasculitis-associated PCT “false-positivity”. Single dose Rux 7.5 mg was initiated but treatment was limited due to progressing multiorgan failure. Patient #5 died on palliative care day 17 after Rux onset.

**Fig. 3:**
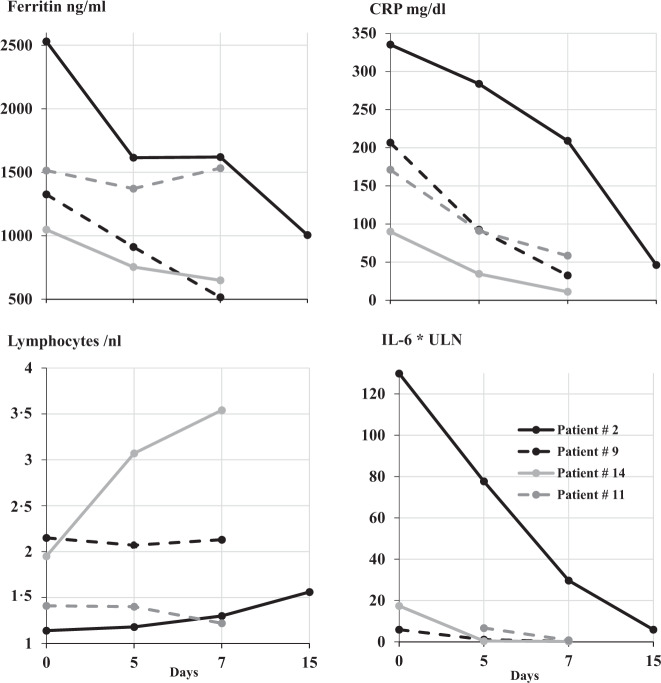
Suppression of inflammation by Rux in patients without concomitant corticosteroid treatment. ULN upper limit of normal.

Patient #2 showed marked clinical and inflammatory response on dose escalated Rux and was weaned to O_2_-supply (Fig. 3).

As the majority of patients was treated with short course of 2 mg/kg prednisolone over three days, we asked, whether Rux treatment can demonstrate CIS-inhibition with clinical response in the absence of corticosteroids. Four patients with a board-decision for contraindications against CS (patients #2, #9, #14) or with a CS-free treatment window of 8 days (patient #11) were put on Rux with marked clinical and CIS-response. Figure 2 demonstrates laboratory response for CRP and IL-6 with variable dynamics of ferritin and lymphocytes in all CS-free treatment episodes, indicating CS-independent anti-inflammatory activity of Rux in COVID-19 induced CRS.

### Toxicity and safety

**Fig. 4:**
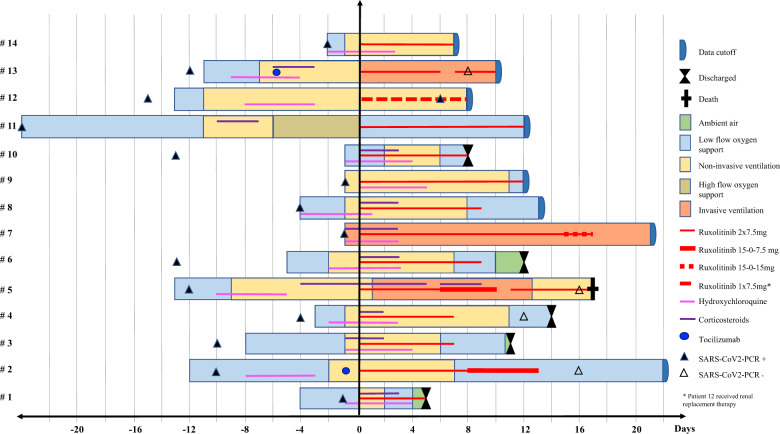
Individual patient tracking with treatment applied and clinical course after Rux onset. Individual patients (n = 14) are depicted on the y-axis, treatment duration is shown on the x-axis (days).

Ruxolitinib has potential for drug induced liver damage, impairment of hematopoiesis and uncertainty with regards to infection control due to reports of viral/bacterial reactivation in patients with MPN [28]. As demonstrated in Table 4 by serial laboratory assessment, liver dysfunction as indicated by increased transaminases was moderate with one patient hitting transient grade 3 liver toxicity. Anemia grade 3 was seen in two patients with pre-existing anemia and treated on ICU with repetitive need for blood drawing. Viral clearance as assessed by soar swabs was checked for anecdotally with four patients tested negative for SARS-CoV-2 while on or after Rux treatment (Fig. 4). One patient showed continuous SARS-CoV-2-positivity. She has been previously immunosuppressed and on renal replacement therapy for vasculitis. In summary, short term toxicity assessment using special interest side effects did not show red flag signals in this limited number of patients.

## Discussion

COVID-19 is a RNA-virus-induced airborne infection with significant morbidity and mortality and no proven antiviral standard therapy. The current pandemic is a global health threat leading to collapse of health care systems even in the most developed countries due to bulk incidence of patients with respiratory failure. Vaccines are in development. Virus directed treatment is under investigation with preliminary efficacy signals in hitherto published trials [2, 3]. Combined antiviral/immunomodulatory treatment with hydroxychloroquine in some countries is a so to say standard with theoretical, but clinically not confirmed activity against COVID-19 [29, 30]. After the disaster of ICU over flooding patients in need of ventilators in Wuhan, Europe, or the USA, it became clear that a subfraction of severely affected patients show hyperactivated T- cells in the peripheral blood and the lungs with systemic hyperinflammation [4–7, 31] stimulating the hypothesis of virus-induced macrophage activation syndrome (MAS) or HLH as potential causes for the consecutive multiorgan failure [19, 20]. Pointing into the same direction are considerations to specifically target the cytokine storm through anti-IL6 or anti-TNF directed antibodies [32–34]. We have not seen HLH/MAS-HLH in our series, as not a single patient showed bicytopenia nor extreme values for ferritin, two criteria with high HLH-sensitivity [35]. We therefore argue for a note of caution against therapeutic actionism targeting classical HLH in COVID-19 using T-cell depleting etoposide [19].

We selected Rux for CRS-targeting due to its record in highly inflammatory conditions such as primary myelofibrosis, HLH, and GvHD [11, 12, 14]. The challenge of managing severely affected COVID-19 patients is the need to coordinate treatment between the COVID-ward medical team monitoring patients at risk for deterioration and to integrate subspeciality knowledge into ICU-management. In order to safeguard detection of patients at risk to develop a hyperinflammatory state, we developed the CIS as a predefined semiquantitative measure for CRS-directed treatment allocation with corticosteroids and/or Rux. This allowed us to develop a COVID-board learning curve for all team members in trying to differentiate COVID-specific inflammation from other causes of IL-6-driven inflammation (sepsis, superinfection). In an emergency treatment situation without available standard treatments and prior to activated clinical trial protocols, this interdisciplinary screening approach can enable medical teams to identify patients with a balanced risk-benefit ratio for novel treatment under investigation. Rux treatment was associated with meaningful inflammation control as assessed by CIS response on day 5 and day 7 in 12/14 pts that correlated with clinical response in 11 out of 14 patients (Fig. 1). Of note, this response was also shown to be achieved without concomitant CS treatment (4/14 pts) (Figs. 2 and 3). Devoid of systematic serial virus testing, we still have some indication of clinical recovery in 4 of the 14 patients with viral clearance despite the fact that Rux has been reported to have the potential of affecting virus control [28]. In this regard, experimental and clinical data suggest JAK1-dependent inhibition of T-cells by Rux explaining observed reactivation of atypical infections in patients on longterm ruxolitinib in MPN [18]. Our approach of short term Rux in severe Covid-19 is intented to stop detrimental cytokine signalling thereby preventing further organ damage. Unnecessary prolonged treatment was avoided to balance the need for calming the cytokine storm against the risk to provoke virus reactivation. Similarly, patients in virus-induced HLH are recommended not to receive extended duration of immunosuppression in order to allow viral clearance [36]. Side effects of short term Rux treatment are well manageable and show the expected spectrum with mild anemia and liver enzyme elevation.

Dosing without pre-existing evidence is a great matter of uncertainty. We developed our dosing guidelines along trials with Rux in GvHD with trial data suggesting good efficacy/toxicity relationship between 5 mg and 10 mg bid [14, 25], and in HLH with individual case reports and a phase I trial providing dose ranges between 5 mg bid and 25 mg bid [12, 13, 37]. Serial efficacy/toxicity assessment in the COVID-board was performed to trigger dose adaptation due to toxicity, or efficacy considerations. The results of treatment have led to the design of a clinical trial with a Rux dose of 10 mg bid and potential dose increase up to 20 mg bid depending on CIS response on days 3, 5, and 7 (NCT04338958) based on the conclusion that our small case series provides us with surrogate and clinical efficacy in patients treated with 7.5 mg bid, but failing in patients critically ill, deteriorating while on Rux treatment without strong signals of toxicity.

Several clinical trials are underway to test Rux in COVID-19. Questions asked are: Is low dose (5 mg bid) compared to placebo efficacious in preventing moderately affected patients from progression to severe/critical disease stages (NCT04362137). Is Rux treatment able to show efficacy in severely affected patients on invasive ventilation due to ARDS (NCT04359290)? A Canadian trial using 10 mg bid is addressing the potential cytokine flare [38] when taking patients off Rux from full dose to zero and directs tapered treatment discontinuation (NCT04331665).

The majority of COVID-19 patients show benign disease course where patients overcome viral inflammation by robust but not overreactive immune response [39]. Treating the majority of those could lead to significant overtreatment while achieving the goal of preventing patients from deteriorating into hyperinflammation. Treating patients on invasive ventilation for ARDS according to our series is likely to be most challenging as irreversible organ damage may be in place and secondary infections cross-react with IL-6/CRP-monitoring of Rux-response. This will require extended and probably dose increased treatment. Treatment in very advanced disease may fall short in preventing the ARDS-associated CRS from causing fatalities, yet this needs to be shown.

Rux is hypothesized to interfere with the detrimental CRS governed by pulmonary inflammation through interference with multiple pro-inflammatory cytokines via JAK-STAT-inhibition. In addition, it may act through antiviral activity by impairing viral replication through interaction with senescence regulation pathways [40]. We are currently in a period with highly vivid hypothesis driven, but low level evidence based clinical emergency management of COVID-19 patients due to lack of proven therapeutics. Our small pilot series has established a reproducible score (CIS) to stratify treatment, which will be tested in our recently initiated RuxCoFlam phase II trial (NCT04338958). To capture individual patients in need for tailored anti-inflammatory treatment without putting too many of them at risk of unnecessary overtreatment, hospital wide education, standardized and interdisciplinary structured treatment and implementation of clinical trials seem to be pivotal.
